# Sérologie palustre: quel apport dans un pays d’endémie palustre comme la Côte d’Ivoire?

**DOI:** 10.11604/pamj.2017.26.221.11314

**Published:** 2017-04-24

**Authors:** Amah Patricia Victorine Goran-Kouacou, Gonat Serge Dou, Kalou Dibert Zika, Adjoumanvoulé Honoré Adou, Oppong Richard Yéboah, Rita Ahou Aka, Sansan Hien, Kouabla Liliane Siransy, Koffi N’Guessan, Tariam Agnès Djibangar, Séry Romuald Dassé, Koffi Daho Adoubryn

**Affiliations:** 1 Département d’Immunologie, UFR Sciences Médicales d’Abidjan, Université Félix Houphouët-Boigny; 2 Département de Parasitologie, UFR Sciences Médicales d’Abidjan, Université Félix Houphouët-Boigny; 3 Département de Parasitologie, UFR Sciences Médicales de Bouaké, Université Alassane Ouattara

**Keywords:** Paludisme, sérologie palustre, Côte d´Ivoire, Malaria, malaria serology test, Ivory Coast

## Abstract

**Introduction:**

La sérologie palustre semble avoir peu d’intérêt dans les pays d’endémie comme la Côte d’Ivoire. Cependant cet examen a été régulièrement réalisé au laboratoire de Parasitologie de l’Unité de Formation et de Recherche Sciences Médicales d’Abidjan. Le but de notre étude était d’apprécier l’apport de la sérologie palustre dans notre contexte de pays endémique.

**Méthodes:**

Nous avons réalisé une étude rétrospective portant sur la sérologie palustre qui a utilisé le kit Falciparum spot-IF de Biomérieux à la recherche d’anticorps antiplasmodiaux d’isotype IgG. Elle a concerné les sérologies réalisées de janvier 2007 à février 2011 et dont les résultats étaient disponibles dans le registre.

**Résultats:**

Au total, 136 patients ont été sélectionnés. L’âge moyen était de 36,3 ans avec des extrêmes de 1 an et 81 ans et un sex-ratio de 0,97. Les indications de sérologie palustre étaient variées, dominées par la splénomégalie (49,3%), les cytopénies (14,7%), la fièvre d’origine indéterminée (13,2%). La quasi-totalité des patients (98,5%) avaient des anticorps antiplasmodiaux avec un titre moyen élevé à 1057,35UI/ml. Il n’existait pas de lien entre l’âge et le titre d’Ac qui était plus élevé pour les cytopénies, les fièvres prolongées et la splénomégalie.

**Conclusion:**

La sérologie palustre a peu d’intérêt dans notre pratique courante en zone d’endémie car quelque soit le motif de la prescription, les titres étaient élevés.

## Abstract (en)

**Introduction:**

Malaria serology test seems to have attracted very little interest in endemic countries such as Ivory Coast. However, this examination has been regularly performed in the parasitology laboratory at the Training and Research Unit of Medical Sciences in Abidjan. Our study aimed to highlight the contribution of malaria serology test in our endemic country context.

**Methods:**

We conducted a retrospective study of malaria serology test using Falciparum-Spot IF (bioMerieux) kit for the detection of IgG antiplasmodial antibodies. It included all malaria serology tests performed from January 2007 to February 2011 and whose results were available in the registry.

**Results:**

In total, 136 patients were selected. The average age of patients was 36,3 years, ranging from 1 to 81 years, and sex ratio was 0,97. Indications for malaria serology test were varied and dominated by splenomegaly (49.3%), cytopenias (14.7%), indeterminate fever (13.2%). Almost all of the patients (98.5%) had antiplasmodial antibodies with high medium titer of 1057,35IU/ml. There was no link between age and Ab titer, which was higher in cytopenias, prolonged fevers and the splenomegaly.

**Conclusion:**

Malaria serology test seems to have attracted very little interest in routine clinical practice provided in our endemic area because, whatever the reason of the prescription, titers were high.

## Introduction

La lutte contre le paludisme, première endémie parasitaire, au niveau mondial est l’une des plus grandes réussites en matière de santé publique de ce millénaire [1]. En effet, entre 2000 et 2015, l’incidence du paludisme a reculé chez les populations exposées de 37% à l’échelle mondiale tandis que le taux de mortalité a baissé de 60%. On estime que 6,2 millions de décès dus au paludisme ont été évités dans le monde depuis 2001[1]. Ces chiffres montrent de toute évidence que la cible 6C des Objectifs du Millénaire pour le Développement « avoir maîtrisé, d’ici 2015, le paludisme et d’autres grandes maladies, et commencé à inverser la tendance actuelle » a été atteint. Cependant, il reste beaucoup à faire. La population exposée est estimée à 3,2 milliards de personnes et plus de 400 000 personnes meurent chaque année à cause de cette maladie évitable [1]. L’Afrique continue de payer le plus lourd tribut à cette endémie parasitaire puisque la plupart des cas (89%) et des décès (91%) au niveau mondial y sont enregistrés [2-5]; les enfants de moins de 5 ans représentant plus de deux tiers des décès [1]. En Côte d’ivoire, le paludisme constitue un problème majeur de santé publique [4]. En effet, 100% de la population y sont exposés au risque du paludisme avec un niveau d’endémicité allant de la méso à l’hyper-endémie [3, 4]. Le paludisme reste la première cause de mortalité (10%) et de morbidité (40%) dans la population générale. Les enfants âgés de moins de 5 ans et les femmes enceintes sont les plus touchés. En outre, la Côte d’Ivoire fait partie des 15 pays dans lesquels la baisse de l’incidence du paludisme a été plus faible que dans les autres. Avec l’avènement des Combinaisons Thérapeutiques à base d’Artémisinine (CTA) dans les protocoles de traitement, la confirmation biologique devant tout cas suspect de paludisme est devenue une nécessité voire une exigence. Ce diagnostic biologique repose sur plusieurs examens notamment la goutte épaisse, le frottis sanguin, les Tests de Diagnostic Rapide (TDR) et éventuellement sur la sérologie palustre [6]. Cette dernière technique, selon la littérature, a peu d’intérêt dans les pays d’endémie palustre tels que la Côte d’Ivoire sauf pour certaines formes cliniques chroniques tel le paludisme viscéral évolutif et la splénomégalie palustre hyperimmune au cours desquelles les anticorps sont à des taux élevés alors que les recherches parasitologiques sont le plus souvent négatives [7]. Ce faible intérêt de la sérologie palustre en zone d’endémie est dû à la cinétique des anticorps antiplasmodiaux. En effet, l’apparition des anticorps antiplasmodiaux est tardive et leur présence peut témoigner soit d’une infestation palustre évolutive soit d’une infestation antérieure dans la mesure où ces anticorps peuvent persister 2 à 3 ans après la maladie [6]. Cependant, la sérologie palustre a été régulièrement réalisée au Laboratoire de Parasitologie de l’Unité de Formation et de Recherche Sciences Médicales Abidjan (UFRSMA) sur demande des cliniciens. Après plusieurs années de réalisation, il nous a paru important de mener cette étude afin apprécier l’intérêt de cette sérologie dans notre contexte d’endémie palustre.

## Méthodes

**Matériel:** Cette étude a été menée à Abidjan, capitale économique de la Côte d’Ivoire. La ville d’Abidjan compte plus de 4 millions d’habitants avec une multitude de quartiers précaires dépourvus d’assainissement (mauvaise gestion des ordures ménagères, des eaux usées, système de drainage inadéquat des eaux pluviales. Les facteurs de risque de transmission du paludisme y sont donc très importants [3]. Il s’agissait d’une étude transversale rétrospective portant sur la sérologie palustre. Elle a été réalisée au laboratoire de Parasitologie de l’UFRSMA et a concerné la période allant de janvier 2007 à février 2011. Ont été inclus, les patients, de tout âge et de tout sexe, ayant eu recours au laboratoire de Parasitologie de l’UFRSMA pour la réalisation d’une sérologie palustre au cours de la période d’étude et dont les résultats étaient disponibles dans le registre dédié à cet effet.

**Analyse des échantillons de sang:** Dans cette étude, la sérologie palustre a été faite sur des prélèvements sanguins réalisés dans des tubes sans anticoagulant à l’aide du kit Falciparum-Spot IF de bioMérieux^®^. Il s’agit d’un test de sérodiagnostic du paludisme à *Plasmodium falciparum* par immunofluorescence indirecte (IFI) dans le sérum humain qui permet de mettre en évidence des Immunoglobulines G (IgG) antiplasmodiums. Les manipulations et les interprétations des résultats ont été faites selon les consignes du fabricant. Ainsi : Titre du sérum : inverse de la plus grande dilution montrant encore une réaction positive; Titre < 20 : absence de paludisme évolutif à *P. falciparum*; Titre 20 ou 40 : infection palustre probablement ancienne ou éventuellement paludisme évolutif à *P. malariae* ou *P. vivax*. Nécessité de répéter les examens à 15 jours d’intervalle; Titre ≥80: paludisme récent ou encore évolutif.

**Recueil et analyse des données:** Le recueil des données a été fait par une fiche d’enquête anonyme élaborée à cet effet. Les données recueillies prenaient en compte les aspects socio-démographiques, les indications ou renseignements cliniques et les résultats de la sérologie palustre avec les différents titres des anticorps antiplasmodiaux. Les logiciels Excel 2007 et Epi-info 2003 ont servi à l’analyse des données. Le seuil de significativité pour comparer deux proportions a été fixé à une valeur P ≤0.05. Les résultats ont été présentés sous formes de tableaux et de figures.

## Résultats

**Figure 1:**
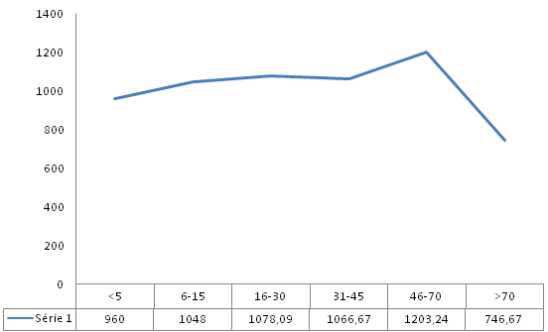
Répartition de la moyenne des titres d’anticorps IgG antiplasmodiaux en fonction de l’âge

Au total, 136 patients ont été sélectionnés dans notre étude. L’âge moyen des patients étaient de 36,3 ans avec des extrêmes de 1 et 81 ans. Les patients de sexe féminin étaient les plus nombreux avec un sex-ratio de 0,9. Le Tableau 1 présente les caractéristiques sociodémographiques. Les motifs de prescriptions de la sérologie palustre par les cliniciens étaient dominés par la splénomégalie (49,3%) (Tableau 2). Concernant les données biologiques, la sérologie palustre était positive (titre ≥80) pour 134 patients soit dans 98,5% des cas. La majorité des patients (113 soit 83,1%) avaient un titre d’Ac antiplasmodiaux compris entre 320 et 1280. Le titre moyen était de 1057,35. Le Tableau 3 expose la répartition des patients selon le titre des anticorps. Le titre des Ac n’était pas lié à l’âge (p = 0,653). Cependant, il était observé une croissance régulière de la moyenne des Ac antiplasmodiaux en fonction de l’âge jusqu’à 70 ans (Figure 1). Les renseignements cliniques ayant les titres moyens d’Ac les plus élevés étaient les cytopénies (4297), suivis de la fièvre (1700), le contrôle après traitement (1600) et la splénomégalie (1344). Mais tous les titres moyens étaient élevés quel que soit le renseignement clinique (Tableau 4).

**Tableau 1:** Répartition des patients selon les données socio-démographiques

*Age* (années)	Effectif	Pourcentage(%)
<5	9	6,6
6-15	20	14,7
16-30	21	15,5
31-45	24	17,6
46-70	37	27,2
>70	9	6,6
**Non précisé**	16	11,8
**Sexe**		
F	69	50,7
M	67	49,3

**Tableau 2:** Répartition des patients selon les motifs de prescription de la sérologie palustre

Motifs	Effectif	Pourcentage
Splénomégalie	67	49,3
Cytopénies*	20	14,7
Fièvre indéterminée	18	13,2
Hépatomégalie	17	12,5
Contrôle post-traitement antipaludique	6	4,4
Paludisme à répétition	5	3,7
Autres**	13	9,6

* **Cytopénies** : anémie (8), thrombopénie (1), bicytopénie (7),pancytopénie (4)

** **Autres :** paludisme viscéral (1), néphropathie glomérulaire (2), insuffisance rénale (1), lymphangite (1), cirrhose (1), cytolyse hépatique (1), céphalées (2), asthénie (3), abcès du poumon (1).

**Tableau 3:** répartition des patients selon le titre des anticorps (IgG anti-plasmodiums)

Titre	Effectif	Pourcentage
0	1	0,7
40	1	0,7
80	3	2,2
160	3	2,2
320	39	28,7
640	52	38,2
1280	22	16,2
2560	7	5,1
5120	6	4,4
10240	2	1,5
Total	136	100,0

**Tableau 4:** répartition des moyennes des titres des anticorps (IgG anti-plasmodiums) en fonction des renseignements cliniques

Renseignements cliniques	Moyenne des titres
Splénomégalie	1344,5
Hépatomégalie	865,9
Fièvre au long cours	1700
Cytopénies	4297,1
Contrôle post-traitement	1600
Paludisme à répétition	1280
Autres	588,6

## Discussion

**Données socio-démographiques (âge et sexe):** L’âge a été mentionné pour seulement 120 patients sur 136 soit 88,2% des cas. Les données manquantes font partie des limites de la plupart des études rétrospectives dont la nôtre. Cela pose aussi le problème du remplissage correct des bulletins de demande d’analyses médicales et de la bonne tenue des registres et dossiers de malades. Tous les renseignements demandés sur les bulletins d’analyse étant importants pour une interprétation correcte des résultats d’analyses et donc pour la prise en charge des patients, il convient d’attirer l’attention des cliniciens et de l’ensemble des personnels de santé. L’âge moyen était de 36,3 ans avec des extrêmes de 1 an et 81 ans. Cependant, les personnes de tout âge ont été retrouvées dans notre étude; cela pourrait s’expliquer par le fait que la Côte d’Ivoire étant une zone d’endémie stable, toutes les personnes qui y vivent sont exposées au paludisme même si les enfants et les femmes enceintes sont les plus vulnérables [8]. Cette même raison peut expliquer que dans notre étude, il y ait presqu’autant d’hommes que de femmes avec un sex-ratio de 0,9. Il est aussi démontré dans la littérature que le paludisme n’est pas une maladie liée au sexe [9, 10].

**Données clinico-biologiques: les motifs de prescription de la sérologie palustre** par les cliniciens étaient dominés par la splénomégalie (49,3%). Cela serait dû au fait que le paludisme à travers ces formes chroniques (splénomégalie palustre hyper-immune et paludisme viscéral évolutif) peut être responsable de splénomégalie ; et dans ce cas, la sérologie palustre offre un argument pour retenir ce diagnostic. En effet, dans ces formes, la recherche parasitologique est souvent négative alors que les titres des anticorps antiplasmodiaux sont très élevés [1]. En dehors de la splénomégalie, les autres renseignements cliniques étaient multiples et variés (confère tableau II). Cependant, certains renseignements cliniques ne sont pas en rapport avec le paludisme. Cette variété de motifs de demande de la sérologie palustre par les cliniciens laisse transparaître une méconnaissance de cet examen, de son apport réel dans la prise en charge des patients et de ses limites. Il y a donc nécessité de formation pour les professionnels de santé. La positivité de la sérologie palustre était de 98,5%. La quasi-totalité des patients (134/136) avaient des anticorps antiplasmodiaux. Cette très forte séroprévalence est due au fait que la Côte d’Ivoire étant une zone d’endémie stable, les populations ivoiriennes sont très vite et très régulièrement en contact avec les plasmodiums. Ce contact régulier permet aux anticorps de persister chez ces populations. Cette même tendance est relevée dans la littérature [7, 11, 12]. Par ailleurs, Diop et al., dans une étude au Sénégal sur la prévention du paludisme post-transfusionnel en zone d’endémie ont trouvé également une forte séroprévalence des anticorps antiplasmodiaux (65,3 %) chez les donneurs de sang [2]. Le kit Falciparum-Spot IF de bioMérieux^®^ permet de mettre en évidence *Plasmodium falciparum*. Cette séroprévalence de 98,5% corrobore les données de la littérature selon lesquelles *Plasmodium falciparum* est l’espèce prépondérante en Côte d’Ivoire [8].

Dans notre travail, le titre des anticorps (Ac) antiplasmodiaux variait entre 40 et 10240. La majorité des patients (113 soit 83,1%) avaient un titre compris entre 320 et 1280. Les titres élevés semblent être caractéristiques des pays endémiques. En effet, selon la littérature, le titre des anticorps est proportionnel à l’intensité et à la durée de l’infestation [13]. En Guinée Conakry, dans une étude réalisée chez les femmes dans le post-partum, Sylla constatait des titres d’Ac également élevés variant entre 80 et 10240 avec une médiane à 1280 [14]. Le titre moyen était de 1057,35. On observe une croissance régulière des moyennes des Ac antiplasmodiaux en fonction de l’âge puis une baisse à partir de 70 ans. Bien que la différence ne soit pas statistiquement significative, cette évolution s’expliquerait par le fait que l’immunité de prémunition antipalustre est relativement faible dans les premières années de vie et chez les sujets âgés. Chez les enfants, cela pourrait s’expliquer par une immaturité du système immunitaire. Cette immunité se consolidant avec l’âge et avec les multiples réinfestations, les titres semblent plus élevés chez les adultes. Chez le vieillard, la défaillance sénile du système immunitaire expliquerait cette baisse de l’immunité de prémunition. Cependant, toutes les moyennes des titres sont élevées (≥640) quel que soit l’âge ; ce qui rejoint l’assertion selon laquelle la Côte d’Ivoire est une zone de haute transmission du paludisme [8].

Les titres moyens d’Ac en fonction des renseignements cliniques montraient que ces titres étaient plus élevés pour les cytopénies (4297,14), les fièvres prolongées, le contrôle après traitement et la splénomégalie. Cependant, pour tous les renseignements cliniques, les moyennes des titres d’Ac étaient très élevées. Par conséquent, la sérologie palustre ne permettait pas de distinguer entre un éventuel paludisme et/ou ses complications et les autres pathologies. Dans l’étude de Diop et al au Sénégal, 65,3% des patients avaient des Ac antiplasmodiaux alors que seulement 0,53% avait une microscopie positive [2]. En outre, même si la littérature parle de l’intérêt de la sérologie palustre dans la splénomégalie palustre hyperactive et le paludisme viscéral évolutif, il est important de signaler que cet intérêt est limité [1]. En effet, la positivité de la sérologie palustre dans ces 2 affections ne représente qu’un argument parmi tant d’autres qu’il faut associer pour retenir ces diagnostics ; en plus dans notre contexte, les titres sont déjà très élevés dans la population générale. Cette sérologie ne saurait donc être d’un grand intérêt. Cet état de fait nous permet de soulever la question de l’intérêt de la sérologie palustre dans les pays où l’endémie est stable. Il est donc nécessaire de sensibiliser les cliniciens sur les limites de cet examen dans notre pratique médicale en zone d’endémie et surtout pour ne pas augmenter les dépenses de santé des patients.

## Conclusion

La quasi-totalité des patients avaient une sérologie palustre positive avec des titres fortement élevés dans la majorité des cas. Et quelque soit le motif pour lequel la sérologie avait été demandée, les titres des Ac antiplasmodiaux étaient élevés ne permettant pas ainsi de distinguer le paludisme et/ou ses complications des autres affections dans notre pratique courante en zone d’endémie. La sérologie palustre est donc d’intérêt limité dans la pratique médicale courante en zone d’endémie palustre.

### Etat des connaissances actuelle sur le sujet

- Dans les pays d’endémie palustre, il existe, une immunité de prémunition du fait des réinfestations fréquentes;
- La présence des Ac antiplasmodiaux témoigne soit d’une infestation palustre évolutive soit d’une infestation antérieure et peuvent persister 2 à 3 ans après la maladie;
- Selon la littérature, le dosage de ces Ac a peu d’intérêt dans les pays d’endémie palustre sauf pour certaines formes cliniques chroniques au cours desquelles les anticorps sont à des taux élevés, ou dans les enquêtes épidémiologiques.

### Contribution de notre étude à la connaissance

- Quelque soit la forme clinique, la sérologie palustre est positive à un titre élevé dans notre contexte d’endémie palustre;
- D’où la question qu’on se pose de savoir si les praticiens devraient demander cet examen dans leur pratique courante.

## References

[1] OMS/UNICEF (2015). Atteinte de la cible des OMD pour le paludisme : inversion de la tendance entre 2000 et 2015.

[2] Diop S, Ndiaye M, Seck M, Chevalier B, Jambou R, Sarr A, Dièye TN, Touré AO, Thiam D, Diakhaté L (2009). Prévention du paludisme post-transfusionnel en zone d’endémie. Transf Clin Biol..

[3] Kouadio AS, Cissé G, Obrist B, Wyss K, Zingsstag J (2006). Fardeau économique du paludisme sur les ménages démunis des quartiers défavorisés d’Abidjan, Côte d’Ivoire. VertigO-la revue électronique en sciences de l’environnement.

[4] Côte d’Ivoire, Ministère de la Santé et de l’Hygiène Publique (2004). Programme national de lutte contre le paludisme: rapport d’activités.

[5] Tagny TC, Mbanya D, Garraud O, Lefrère JJ (2007). Sécurité transfusionnelle: paludisme et don de sang en Afrique. Transf Clin et Biol..

[6] Siala E, Ben Abdallah R, Bouratbine A, Aoun K (2010). Actualités du diagnostic biologique du paludisme. Rev Tunis Infectiol..

[7] Wong J, Hamel MJ, Drakeley CJ, Kariuki S, Shi YP, Lal AA, Nahlen BL, Bloland PB, Lindblade KA, Were V, Otieno K, Otieno P, Odero C, Slutsker L, Vulule JM, Gimnig JE (2014). Serological markers for monitoring historical changes in malaria transmission intensity in a highly endemic region of Western Kenya, 1994-2009. Malar J..

[8] Côte d’Ivoire, Ministère de la Santé et de l’Hygiène Publique (2008). Programme national de lutte contre le paludisme. Directives de prise en charge du paludisme.

[9] Reuben R (1993). Women and Malaria-special risks and appropriate Control Strategy. Social Science and Medicine..

[10] Tin-Oo, Pe-Thet-Htoon, Khin-Thet-Wai, Will Parks, Joan Bryan (2001). Gender, mosquitoes and malaria: implications for community development programmes in Laputta, Myanmar. Southeast Asian J Trop Med Public Health..

[11] Cunha MG, Silva ES, lveda NS, Costa SPT, Saboia TC, Guerreiro JF, Póvoa MM, Corran PH, Riley E, Drakeley CJ (2014). Serologically defined variations in malaria endemicity in Para State, Brazil. PLoS One..

[12] Diallo P, Keita N, Barry I, Condé NM, Diallo PM, Sylla A, Lamah OO, Camin AM, Guiguen C, Sénécal J (1998). Impaludation du nourrisson dans une zone rurale de Guinée maritime (Guinée Conakry): II - Evolution des anticorps antipaludéens et impaludation au cours de la première année de vie. Parasitologie.

[13] Chippaux JP, Du Saussay C, Akogbeto M (1989). Intérêt du dosage des anticorps palustres chez les sujets non immuns en zone holoendemique. Médecine tropicale..

[14] Sylla A, Lamah OO, Camin AM, Diallo P, Keita N, Barry I, Conde NM, Diallo PM, Guiguen C, Sénécal J (1998). Impaludation du nourrisson dans une zone rurale de Guinée maritime (Guinée Conakry): I- Statuts immunitaire et parasitaire de la mère et du nouveau-né. Parasitologie.

